# No paper, but the same routines: a qualitative exploration of experiences in two Norwegian hospitals deprived of the paper based medical record

**DOI:** 10.1186/1472-6947-8-2

**Published:** 2008-01-10

**Authors:** Jan-Tore Lium, Aksel Tjora, Arild Faxvaag

**Affiliations:** 1 Department of Industrial Economics and Technology Management, and Norwegian Research Centre for Electronic Patient Records, Norwegian University of Science and Technology (NTNU), Norway; 2 Department of Sociology and Political Science, and Norwegian Research Centre for Electronic Patient Records, Norwegian University of Science and Technology (NTNU), Norway; 3 Norwegian Research Centre for Electronic Patient Records, Faculty of medicine, Norwegian University of Science and Technology (NTNU), Norway

## Abstract

**Background:**

It has been shown that implementation of electronic medical records (EMR) and withdrawal of the paper-based medical record is feasible, but represents a drastic change in the information environment of hospital physicians. Previous investigations have revealed considerable inter-hospital variations in EMR system use and user satisfaction. The aim of this study was to further explore changes of clinicians' work after the EMR system implementation process and how they experienced working in a paper-deprived information environment.

**Methods:**

Qualitative study based on 18 semi-structured interviews with physicians in two Norwegian hospitals.

**Results:**

Ten different but related characteristics of work within the EMR-based practice were identified; (1) there was closer clinical and administrative cooperation during the implementation processes; (2) there were greater benefits when everybody used the system; (3) systems supported freshmen better than experienced physicians; (4) the EMR was useful in regard to professional learning; (5) new users were given an introduction to the system by experienced; (6) younger clinicians reported different attitudes than senior clinicians, but this might be related to more than age and previous experience with computers; (7) the EMR made it easier to generate free-text notes, but this also created a potential for information overflow; (8) there is little or no support for mobile work; (9) instances of downtime are still experienced, and this influenced the attitude towards the system and (10) clinicians preferred EMR-only compared to combined paper and electronic systems.

**Conclusion:**

Despite the removal of paper-based records from clinical workflow (a change that hospital clinicians perceived as highly useful), many of the old routines remained unchanged, limiting the potential of the EMR system. Thus, there is a need to not only remove paper in the physical sense, but also to established routines to fully achieve the benefits of an EMR system.

## Background

For decades the electronic medical record (EMR) has been described as having the potential to increase both quality and efficiency of health care delivery [1]. In many countries, EMR systems are however not widely disseminated. Furthermore, EMR system vendors appear to face almost the same challenges now as decades ago [2]. In Norway, as well as in some other small European countries, most general hospitals have finally implemented and started to use EMR systems. For these hospitals, there is now a continuous struggle to realize the expected and desired benefits of EMR's, mainly related to removing its paper-based ancestor and changing the time-consuming routines that were necessary as long as the records were physical paper folders. A national cross-sectional study about hospital physicians' use of EMR's revealed that substantial proportions of the available EMR system functionality were not used by the physicians [3]. A possible explanation being the fact that the EMR systems have existed in parallel with paper based medical records, leaving the physicians to choose which medium to use.

After a change in regulations in 1999 that allowed for the possibility for hospitals to replace their paper archives with EMR's, several Norwegian hospitals embarked on a process to become paperless. Based on our studies of EMR system implementations in Norwegian hospitals [4], this can be described as a four stage process (table 1).

**Table 1:** The different stages in eliminating the paper-based medical record

**Stage**	**Paper based medical record**	**Electronic medical record**
I	Present and updated	Absent
II	Present and updated	Present
III	Present, **not **updated	Present, and supplied with **scanned **documents
IV	Absent	Present, and supplied with **scanned **documents

Today only one general hospital in Norway remains at stage I. The majority are at stage II, while a few have eliminated their paper based record and now only archive patient data in an EMR system (stage IV). Some hospitals have gone directly from stage I to IV, others appear to have stalled at stage II, some of these for more than five years [4]. The first hospital to reach stage IV was the subject of a study in 2002. This study revealed large variations between health professionals' use of the EMR system. While medical secretaries reported to be very pleased with, and used the system extensively, physicians and nurses used the system mostly for tasks for which they had no choice but to use the system due to the lack of a paper based record [5,6]. In a 2005 follow-up we found a large increase in EMR system use among physicians and nurses that largely were independent of technological factors, possibly indicating that physicians and nurses had adapted to the EMR system [7].

To broaden our understanding of EMR system implementation processes and the impact of eliminating the paper-based medical record on the work of hospital employees, we extended our study to include physicians, nurses and medical secretaries from six different Norwegian hospitals [4]. This study revealed considerable differences between the various departments/hospitals both with regard to reported use of the EMR system and whether the EMR system eased the performance of clinical tasks. Some departments stood out: Here the physicians both reported an extended use of the EMR system and a positive attitude to the changes imposed on them. In this study we have elaborated further on physicians' use of the EMR system in hospitals deprived of the paper-based medical record. We have conducted semi-structured interviews with hospital physicians from the two hospitals reporting to have the highest use of EMR and inquired about a) which organizational factors that may have led to such a high utilization of the EMR system and b) clinicians' experiences of working in a paperless environment.

## Methods

### The hospitals

We chose to interview physicians from two hospitals reporting to have the higest use and physician satisfaction with the EMR system [4]. Hospital North and South are community hospitals serving a population of about 40.000 and 100.000 respectively. Hospital North, with about 115 beds, is located in northern Norway and Hospital South, with about 245 beds, in southern Norway. Hospital South was also the focus of a previous study published in 2006 [7].

### The EMR systems

Both hospitals had an installation of DIPS-EMR [8], which is both an EMR system and a patient administrative system. The system also supports the ordering of X-ray examinations and laboratory tests, and will accept and store radiology and laboratory reports. Nursing documentation was also electronically implemented at both hospitals. None of the hospitals had a large-scale decision support system. Both departments included in our study had removed their paper-based medical record from clinical workflow. Hospital North and South made this move when they implemented their EMR systems in 2002 and 2001 respectively. The EMR has status as a legal document. Thus, both these hospitals went straight from paper only to EMR only.

### The scanning processes

Since the paper-based medical record is no longer available, historical data have to be included in the EMR by scanning relevant parts of the old paper-based medical record. The scanning processes were very similar in both hospitals involved in our study. Upon admission to the hospital, it is checked whether the patient has an old paper-based medical record. If so, the majority of its content is scanned and made available through the EMR as *scanned multiple documents*. That is, image-files with multiple pages, sorted according to broad categories (table 2).

**Table 2:** Scanning categories. The number of subsections within the categories and the degree of *scanned multiple documents *versus *scanned single document*s varies between the hospitals.

**Category**	**Examples**
Summaries	E.g. Index of consultations and admissions, Discharge reports, Discharge reports from other hospitals.
Textual medical record	E.g. Continuous textual medical record (admission reports, surgery reports etc.), Referrals within the hospital
Lab results – tissue and body fluids	E.g. clinical biochemical/immunol./pharmacol. Investigations
Organ functions (incl. photographs)	E.g. cardiovascular function, lungs and respiratory function
Radiology and other imaging	E.g radiological investigations, CT, MRI, ultrasound.
Treatment, observation and anestesia forms	E.g. patient chart summary and treatment forms.
Nurses' documentation	E.g. nurse's admission reports and notes
Other health personnel	E.g. physical therapist, occupational therapist
Correspondence	E.g. admission request forms
Certificates/notifications	E.g. various public certificates, forms and notifications

In addition to *routine electronic data *(searchable data entered directly into the EMR) and *scanned multiple documents*, an EMR might also contain *scanned single documents*. These are new documents either coming to the hospital in the form of paper (e.g. a report from an external laboratory or a referral letter written by a GP that does not transmit these electronically) or are paper documents produced during the stay (e.g. the medical chart).

### The interviews

18 semi-structured interviews among physicians at the medical departments were conducted in May-June 2006. At Hospital South, 11 physicians were interviewed. 4 interns, 4 residents and 3 seniors. At Hospital North, 1 intern, 3 residents and 3 senior physicians were interviewed. Nurses, physiotherapists and other professionals also use the EMR systems. However, to limit the already broad scope of the study, those were excluded. The interviews, which lasted from about 25 to about 45 minutes, were conducted by the first and third author at Hospital South, and the first author at Hospital North.

The background for the interview study was, as mentioned, a survey that both departments in question had participated in [4,7]. Based on that survey and inspired by literature regarding both EMR usage [3-7] and introduction of information technology more in general [9,10], an interview guide was developed. The structure was limited to the areas we wanted to address (such as the implementation process, how the training was organized, the ease of using the system, functionality that was especially valued or missed etc.), and within these areas the conversation was largely unrestrained. Thus, there were none specific questions and we were also ready to pursue other themes that the respondents brought up during the interviews.

The interviews were taped and later transcribed by the first author. An inductive analysis was supported by the use of QSR NVivo 7 software for qualitative analysis, by sorting out specific themes that occurred in the interview transcripts. That the analysis was inductive means in this case that themes (or nodes in NVivo terminology) were identified in the transcripts regardless of their occurrence in the interview guide, very much influenced by a grounded theory approach [11]. Especially, we emphasised themes that repeated themselves across the respondents. The themes were thereafter matrix-coded and analyzed according to department, the experience of the physicians etc. to identify potential systematic relations in our empirical material. Since our material is fairly limited, our aim has not been to test significance of such relations, but rather to explore physicians' experiences with EMR with their individual background in mind.

## Results

Ten different but related themes could be extracted from the interviews; (1) the implementation processes (what did they do and so on); (2) larger benefits when everybody used the system; (3) the systems supported freshmen better than experienced physicians; (4) EMR is useful in regard to professional learning; (5) seniors standing for the initial system training, new users learning from those with experience, (6) younger users reporting different attitude than seniors, but might be related to more than age and computer experience; (7) easier to produce text, but a potential for information overflow since complex case histories are hard to browse through; (8) little or no support for mobile work, with few or no handheld devices; (9) instances of downtime, which influence the attitude towards the system and (10) aspects regarding the EMR-only situation compared to maintaining dual systems.

The themes that emerged during the interviews were overall very similar at both hospitals. Thus if not otherwise specified in the text, the descriptions apply to both. We will now discuss in more detail these themes.

### The implementation processes

Both hospitals involved in this study had a similar approach to the introduction of their systems, and both went from paper based medical records only to EMR only. Hospital South was the first in Norway to withdraw the paper based medical record from clinical workflow, and Hospital North began their approach by learning from Hospital South.

"I guess we were the hospital that sort of adopted the largest amount of functionality in the shortest period of time. We had been down at Hospital South and looked at what worked and what didn't work down there, and tried not to make the same mistakes" (Hospital North, senior 3)

One important factor for the apparent success, as reported by physicians from both hospitals, was that both clinicians and key personnel from the management were strongly involved and enthusiastic about the project. There was a strong common understanding that the EMR had the potential of becoming a useful tool, and this point of view was broadly communicated throughout the organization.

"The director and chief physician took a lot [of decisions]. It became sort of an enlightened kingdom where decisions were made and then [people in] the rest of the organization were informed why they had been made. [...] So that, during the introduction [of the system], I think it's important that you have a strong and clear leadership that says: 'Boys, this is the way it's going to be!' And then we had to sit down and figure out how to do it". (Hospital South, senior 1)

"It came mostly from me and those who, worked with the introduction of DIPS [to introduce as much functionality as possible...] But we quickly got acceptance when, the department leadership committed to the idea, and that was important for us". (Hospital North, senior 3)

In both hospitals the decisions were mainly made by the chief management and clinical leadership, especially with regard to which system-functionalities should be mandatory, as well as the rationale behind the decisions which was communicated extensively. During these presentations input from staff at all levels was accepted and appreciated.

"Generally, I think the information was good. Especially the [coordinator] and the local IT department did a good job. It's important when you are about to introduce an EMR system that you do like we did here, you strengthen the IT department and, they had plenty of local IT consultants and had an adequate number of administrative resources to do the scanning of the old paper based records". (Hospital North, senior 1)

"We had a marvellous coordinator. She [..] is excellent [and] did a great job. Went everywhere and always kept staff informed and so on. But the decisions.., everybody was involved in the process, but the decisions were made up there [chief level]. And it was very clear that it was the director who made the decisions. And then you could be involved in the processes. So there were very orderly and clear lines [of communication or decision-making?], which I think is very important". (Hospital South, senior 1)

As described above, even if only a few actors were involved in making the initial decisions, involvement and engagement spread among staff as the implementation project continued. Physicians from both hospitals reported that they had good contact with the implementation personnel and local IT departments; project managers were especially praised. The local IT departments were described as having positive attitudes during the process and willingness to go to great lengths to respond to requests from users.

"I think that our IT department, relatively regularly [..] had meetings with us and heard our requests, and we saw that if [these] did not come through we got feedback about why they could not be done. [...] So, we were straightforward with regard to evaluation in the period after introduction, [and it] made people feel that they had some degree of influence". (Hospital North, senior 1)

Physicians received very prompt and thorough feedback if their requests not could be met due to technical issues or because of budget limitations. Thus, they were left with the impression that their inputs where of value and taken into account, even though their suggestions did not always lead to changes in the system. The local IT departments continued to play a vital role also after system launch, in that users got very rapid help if they had any questions regarding use of the system.

Despite very similar approaches to implementation, there were some differences between the two hospitals. A leading physician from Hospital North stated that Hospital South had granted their physicians too much freedom during the implementation process in that it was up to each physician to decide whether or not to use the system for tasks where the previous, paper-based routine was still an alternative (e.g., write prescriptions, fill out paper-based sick-leave forms, etc.). In contrast, Hospital North enforced the change of routines to a larger extent: physicians had no choice but to use the system for the majority of tasks. However, they pointed out some important factors that made it possible for them to do so.

As mentioned above, the physicians at both hospitals were well informed before the systems were implemented. This did not result in all clinicians being overly positive or optimistic about the project, but there was no evidence that ambivalent clinicians made attempts to sabotage the system. One chief physician at hospital North attributed this to their organizational culture. Physicians who did not support a particular view were encouraged to raise their objections and were listened to, but once a final decision was made, they did their best to adapt.

"Well, I guess we didn't have any particular expectations, but maybe that, it would be harder to navigate and more difficult to get an overview and so on, but it's not like that. That's because the records are pretty complete. [...] I have to say it has worked out far better than expected. A lot of the objections we had to begin with have been proved wrong". (Hospital North, senior 2)

As illustrated above, the physician was initially reluctant regarding use of the system, but nonetheless tried to cope with the change and in the end adopted the EMR system as a useful tool.

Another difference between the two hospitals was that Hospital North to a larger extent had combined the EMR system implementation with attempts to change clinical routines to exploit the advantages of electronic workflow. These changes were not very extensive, but as we will come back to in the next section, they were enough to highlight the importance of regarding organizational and technological changes as interrelated issues.

### Greater benefits when everybody used the system

For some system functionality usage varied from department to department, but it also varied between individual physicians in the same department. At hospital South, where physicians had the greatest degree of freedom, use of the system for writing prescriptions or sick-leave notes varied considerably.

"I find it much faster to write prescriptions by hand. And sick leave notes too [...] Can't be compared. It takes half the time". (Hospital South, resident 3)

"The usual stuff like sick leave notes and prescriptions are very easy to write [using the EMR system]. Sick leave notes go very fast, a lot of the information that has to be there comes up [automatically]" (Hospital South, resident 1)

The physicians cited above come from the same department, yet they describe the perceived usefulness of a given functionality in completely opposite ways. Both those in favor of the system and those who preferred pen and paper reported that their method was the most effective. Those who had started to use a particular function in the system reported doing so on their own initiative, whereas those who preferred paper said there was strong organizational pressure on them to start using the EMR system.

At Hospital South, as mentioned in the introduction, there had been a significant increase in the use of these optional functionalities over a three-year time span. The reason, as suggested by the physicians, was a 'natural adaptation' to the system. As the EMR system gradually became an integral part of everyday work, physicians more often got the opportunity to observe other colleagues using the system and to discuss EMR system functionality with their peers. Everyday use of the EMR system also became a learning environment where users' EMR system skills spread between physicians. Some physicians also reported to have benefits from always being logged on to the EMR system. Seen in isolation, a particular task might be easier to do with the use of pen and paper (i.e. logging into the system to conduct the task was more cumbersome), but if one had the EMR on the screen in the first place, the task was much faster to perform with the EMR.

Hospital North, as mentioned above, had a slightly different approach than Hospital South in that it left users with fewer choices regarding whether or not to use the system. These constraints on the freedom of physicians did not have a negative impact on physician satisfaction with the use of the system, as Hospital North physicians were more satisfied with the use of the EMR system compared to their colleagues in South.

"As long as I've been a physician here I've used DIPS so I don't know about other systems at all. I did my internship at another hospital that also had DIPS, but they didn't use all the functionality we do here [at Hospital North]. And it was definitively things I missed at that hospital which I use here. For instance, we didn't enter medications in DIPS and didn't write medical charts notes in the same way" (Hospital North, resident 1)

Accordingly, some of the physicians had very little experience in using tools other than the EMR system, which might also influence their attitudes towards the system. As for the tasks of writing prescriptions and sick-leave notes, physicians from the selected department at Hospital North mentioned one important factor they thought had contributed to the ease of performing these tasks in their department: they used the medication module offered by the EMR system. Despite not having an electronic medical chart available, Hospital North had changed their admittance and discharge routines slightly to take advantage of the possibilities offered by the medication module. The admitting physician at Hospital North always updated the patient's medication list, and a paper medical chart with this information was printed. During the stay the paper-based medical chart was updated, but was scanned upon discharge. Also, during discharge, the medication module was updated once again, and according to physicians, this greatly improved the ease of writing for instance prescriptions and sick leave notes.

"If the medication-stuff had worked it would have been very useful to have it in DIPS you know – which drugs the patient is on – if a new patient is admitted you look at previous arrival notes [...] If you had a system that was continually updated [...] You've got it today, but it doesn't work [...] It's not so good that I trust it [...]. It may have changed, you can't be certain that it's updated". (Hospital South, intern 3)

At Hospital South, the use of the medication module was optional. Only a fraction of the physicians used the module to create and update the patient's medication list. A physician could therefore not rely on the electronic medication list being updated, slowing down the previously mentioned tasks.

As reported from Hospital North, one reason for high EMR usage was that the management required the physicians to use the system and that the physicians obeyed this requirement. By applying an EMR system with a broad range of functions, and knowing that everybody used the system, the hospital achieved a 'use economy of scale. One of the strengths of computer systems is the way they can facilitate re-use of information, particularly when information is represented as structured data. One of the key factors for Hospital North's ability to maintain a high degree of use and satisfaction with the EMR system seemed to be the fact that nearly all information was entered into the system, with the key ingredient being the updated medication list in the medication module.

### The systems supported freshmen better than experienced physicians

Interns described their role, humorously, as being the departments' "medical record slaves". The interns typically have the first contact with the patients, at least for patients in need of emergency treatment, and in that situation the interns often need a lot of information very quickly. They reported great usefulness in having a complete EMR available, where they could quickly accessed notes from previous encounters, discharge reports, and the medication lists from the last hospital stay. In addition, they could order laboratory tests and x-ray examinations from the same system.

Residents also generally had a very positive attitude towards the system. The EMR system helped them gain a better overview of the patients on the ward before they presented them at the morning briefing. As one of the residents said:

"Logging onto the EMR system is the first thing I do when I come to work. To check which patients have arrived at the ward, I can read up on the patients before I go to the morning briefing, read the arrival notes, I can read the nurses report if I want to. [...] Various test results [and] medications I get from the arrival note, but I can check [in the EMR] if I want to" (Hospital North, resident 1)

Senior physicians generally reported the same advantages with the EMR systems as interns and residents. However, they also described tasks that the EMR system not did support, and highlighted that the EMR systems basically was a documentation system with modest support for electronic workflow. Among the many responsibilities of senior physicians are the tasks of assessing referral letters and monitoring the overall medical quality of the department. While senior physicians reported that getting an overview of the patient and evaluating referral letters had become easier with an EMR system with electronic workflow, support for patient logistics was at best modest. Extraction of data for developing quality indicators was also cumbersome. Respondents suggested specific tasks that deserved better support by the EMR system:

"if you eventually could have an electronic system that calculated time needed, and booked automatically [...] x-ray examinations, blood samples, all the things you now have to do manually, when the patients arrived. [.. some sort of] planning tool". (Hospital South, senior 3)

"What annoys me somewhat is that you have very limited influence on the system, and limited ability to generate reports yourself. How many [patients] with [a certain disease] have been admitted this year and so on. It's not good enough. Then you could browse through and, yes, now I've got a question about how many new [diseases] you have had this year. And then I could see, but you can't do that using DIPS, because you don't register if a diagnosis is new or old, you can only see how many we've had with [this disease]. So, a good report generator. That is very much missed by most of us". (Hospital South, senior 1)

The senior physicians emphasized the lack of ability to generate data reports, with which they could, for example, get overviews on diagnoses, complications, procedures performed, etc. These issues were now mainly handled manually or through separate department-specific systems, which were rarely integrated with the EMR. So, while the interns, residents and chief physicians mainly saw the same advantages with a paperless EMR system and electronic workflow, we also got the impression that the more senior and responsible physicians were, the less support they received and the less usefulness they perceived from the EMR system.

### An EMR system is useful in regard to professional learning

All physicians involved in this study regarded their EMR system as useful for professional development, but some functionality was missed. Interns are enrolled in an educational program at the hospital, and both interns and their supervisors concluded that the EMR had improved the assessment of the interns' documentation skills. However, few supervisors systematically used the EMR system to control the interns' documents, both due to time pressures and the fact that they also had regular face-to-face meetings with their interns during which these issues were discussed. If an intern was uncertain of what to do about a particular case, the EMR system made it easy to either call a chief physician who could look up the patient's case history in the EMR, or (in less urgent cases) send an electronic note to a chief physician who would see the memo in his work-list and could look up the case when he had time. Further, if someone needed a specialist to interpret an x-ray image and the physician in question was in the operating theatre, the picture could be displayed on screen in the theatre. So, in the same way as the EMR system was useful when answering external calls about inpatients, it also facilitated electronic communication between professionals within the hospital.

Physicians highlighted two categories of functionality that they especially valued: Collaborative work, at least mediating of questions as described above, and functionality summarizing an individual's or a whole department's work in various reports (i.e., generating data reports). While the first to some degree was supported in the EMR, the latter was supported to a very limited degree at both hospitals. Interns and residents missed the ability to go back in time and read documents of previous patient encounters to recall and learn from interesting cases. Chief physicians missed the ability to generate reports about procedures, complications etc. as described above.

### New users were given an introduction to the system by experienced users

Before the launch of the system, all physicians from both hospitals went to training sessions to be able to efficiently use the system once installed. In addition, there was increased manpower in the support section of the local IT departments during launch. After a while, as the system became more of a part of everyday work at the hospitals, this changed.

"When we arrived, a resident at the surgical department gave us a short introduction to DIPS. This was nice, since a lot of us didn't know anything about the system to begin with. And then it becomes sort of gradual.., you sit next to someone who knows a lot, and suddenly.., things go even smoother [in regard to using the system]". (Hospital South, intern 1)

New users were given a short (between 30 minutes and one hour) introduction to the basic functionality of the system during their first days at the hospital. From then on, they were largely left to learn the system be themselves, but were aided by more experienced users when needed. Still, the physicians we interviewed reported that this arrangement worked adequately. They were quickly able to use the most important functionality, and could deal with the system in a short period of time. However, some physicians said this arrangement made training and the general attitude towards the system somewhat person-dependent. Several interns said they were influenced by how their supervisors and close colleagues used the system, and most of them also pointed out several additional functions in the system that could be useful, but due to time pressures they had not prioritized spending more time learning the system. Some physicians also wanted better follow-up instruction after they had worked with the system for a period of time. For instance, having a system expert joining them in their work a few hours a year, providing them with clues in how to improve their use of the system and suggestions on how to perform tasks they felt cumbersome in other ways. At the same time, this process could help further development of the system by providing valuable feedback to system developers.

### The juniors reported different attitude than seniors

As described above, recently employed physicians usually got an introduction to the basics of the system from more senior physicians. However, when it came to more advanced or optional functionality, most interns and residents reported of a difference in attitude between junior and senior physicians. While all physicians used the EMR systems for tasks where they more or less had no choice (e.g., finding specific information) senior physicians – according to the younger ones – tended not to use the system if they had a choice (e.g. write sick-leave notes or prescriptions). As one intern said:

"We youngsters might catch certain computer issues faster than they do [senior physicians]. That's just the way it is. However, it's nice to have the advantage there compared to a lot of other stuff..". (Hospital south, intern 3)

When it came to learning how to master more advanced use of the EMR system, roles were inverted: The younger, more computer-savvy clinicians assisted and supervised senior clinicians when needed, in a manner identical to senior physicians' assistance and supervision of interns and residents in issues of medicine and healthcare. This did however not seem to bring additional tension in the relation between senior and junior physicians.

### Easier to produce text, but a potential for information overflow

Most respondents reported that it was easier to generate text, such as journal notes, in the EMR system than in the previous paper-based medical records. All respondents reported typing short journal notes, while longer notes (e.g., outpatient department notes and discharge reports) were dictated and later transcribed by medical transcriptionists. Some physicians had also begun to write discharge letters on their own, reusing data from the system to ease the process. Further, the reduced effort of producing text had increased the communication aspect of the medical record. Often, physicians wrote a small note in the EMR system to update the physician scheduled to be on duty the next morning. Thus, more updated information was available, also during weekends, when it could take a long time from the time of dictation to when the notes were transcribed.

"For the patients who have been at the ward for a long time there are a lot of documents named journal notes. And that could be anything from those two lines beginning with [medication] because of this or that, to long comprehensive notes that are very useful. [...] The thing with DIPS is that for patients moving in and out the hospital a lot, it's hard to find what you look for. You get these long lists of notes. And journal notes are hard to find, so then the paper journal might be easier, because you often have a summary up front. And yes, you browse faster through paper compared to opening note after note on the computer, which also might be a slow one..". (Hospital South, intern 2)

"You get a lot of documents for certain patients in DIPS, and then, if you don't know how to filter out a lot of those documents, you can end up spending a lot of time trying to find what you want". (Hospital North, resident 4)

As illustrated above, the increased ease of documenting and the chronological structure of the EMR come at a cost; an increased amount of free text in the medical record. Several informants reported that the increased number of short notes and the lack of structure made it more cumbersome to get an overview of patient cases. The physicians missed the ability to filter out what they regarded as insignificant documents. They wanted to be able to highlight documents that carried important information. For instance, physicians were not generally interested in notes describing that a lab test had been sent to an external laboratory, or in letters to patients reminding them of a forthcoming encounter.

Hence, the respondents wanted better ways to filter documents, or another way of structuring the record so that they could more easily access relevant and important information. In that respect, it was also reported that the use of scanned document images should be kept to a minimum. Images are at the outset not searchable, and therefore more cumbersome to use than electronic data represented as text and numbers. In addition, if the scanned documents are hand-written, they are often more illegible after they are scanned than they were in their original form. So, even though historic data have to be scanned to get a complete EMR, new information should, to a large extent, be entered as searchable data. This was also the case at the time of this study, as physicians reported far less use of scanned documents as time passed by.

### Little or no support for mobile work

Despite electronic workflow and complete EMR systems, the medical records of the departments involved in this study were not completely paper-free. For instance all departments kept a paper-based medical chart that was scanned after patient discharge (the vendor is currently developing an electronic chart). Physicians also regularly printed parts of the EMR to keep some information about the patients in their pockets when they not were in front of a computer. However, printouts of large parts or the whole EMR were very rare.

"Well, it's often, like when you do your rounds on the ward there are several times it would be useful to be able to look at the patient's journal, because you get questions – has that test result arrived? etc. – and you might need to quickly browse through the case history or something...". (Hospital South, intern 4)

"If you have done a proper job in advance you don't need [a mobile medical record]". (Hospital North, senior 2)

As for mobile platforms, both departments had previously tested the use of laptop computers with wireless networking. However, physicians reported that walking around with laptops hindered, rather than, supported their work, so such solutions were rarely used. Also, they said that as long as the medical chart was still at least in part on paper, mobile solutions afforded only modest benefits. They emphasized that a mobile electronic medical chart, which they thought would be introduced shortly, had to be very easy to use and preferably in a format that could be easily carried around. Last, some physicians were skeptical to an electronic version of the medical chart, as the daily operation of the ward then would become even more sensitive to EMR system downtime (that should be non-existent, but nonetheless happens).

### Still instances of downtime

All departments involved in our study had experienced incidents of EMR system downtime. Mostly, these were planned so that the affected departments could be prepared accordingly, but short incidents (e.g., those lasting up to one hour) of unplanned or sudden downtime had also occurred. None of the physicians in our study had experienced patient-related problems associated with system downtime. However, they reported that such instances were very hectic and troublesome, and should be avoided. Also, not having information available might in certain cases jeopardize patient safety, even though it had not been reported as the case so far.

"At nights, if you take those half hours when the EMR system is down and compare it to those nights – which was almost every night – when you run around looking for the paper-based medical record, we are now approaching 100% access to patient information – while you at night with the paper record had maybe 80–90% access. So I think 10–20% of [the] missing information is now reduced to a couple of percents, maybe one. Less I guess". (Hospital North, senior 1)

As demonstrated, physicians frequently lacked access to the medical record in the era of paper-based medical records. Still, downtime was a scary thought to most respondents. Also, as some respondents stated, unplanned system downtime created scepticism towards transferring the medical chart to an electronic format. Hence, the physicians were sensitive to system downtime, but were able to cope with those few and brief instances.

### EMR only versus dual systems

Of the physicians in this study, none had extensive experience working with both a paper-based medical record and an EMR system in parallel (dual systems). Some had experience from other hospitals and senior physicians had experience from the days of the paper records, but interns and residents from these hospitals had mostly worked with EMR only.

"Well, I like to focus on the positive aspects, so I think that life.., by having the information available at any time, that's, it's a completely new life. I mean, as a specialist, in an area with a lot of patients, you get a lot of questions from physicians out there [GP's etc.], and by not having to request the paper medical record, but having the information instantly, it's a totally different situation, a completely new world". (Hospital South, senior 1)

What became clear from the interviews was that no physicians missed the time when they had only paper-based medical records and a paper-based workflow. A few of the residents at Hospital North had some experience with dual systems (EMR system and paper records in parallel), though the EMR systems in question had less functionality. These residents told us that in these periods they missed the all-embracing, highly functional EMR system they were used to, and could not understand how the physicians at other hospitals could be satisfied with their solutions. On the other hand, physicians in these hospitals were used to parallel systems and could not understand why visiting physicians were complaining, as they were quite happy with the way things worked. Still, as physicians from Hospital North and South told us, the real benefits, and the real joy of using the system, was when almost all information they needed could be accessed through the system without the need for multiple logins, everybody were using it and could communicate through the system and trust that the person they were trying to reach also used the system. Even when we challenged them during interviews by arguing for some benefits of the paper (e.g., ease of browsing, familiarity, portability, etc.) most of the physicians did not agree. If they really wanted to, they could always print what they needed. Of the 18 physicians interviewed at Hospital North and South, only one missed having a paper based medical record available, but only during a single patient's stay.

## Discussion

### The effect of strong clinical involvement on the outcome of the implementation processes

When looking at the implementation projects in Hospital North and South, both sought to benefit from the 'traditional' factors of success [12]. These include informing and engaging end users, being aware of and having a strategy for handling organizational resistance, creating ownership at all levels and by all groups, getting support and participation from the management and having a proper training program [9,10,13,14]. Including these factors in project plans does not, however, automatically lead to success. Berg [15] points out that the danger of focusing too strongly on critical success or failure factors in the implementation of a patient care information system (PCIS). Unintended consequences are prone to emerge [16,17], and the danger, according to Berg, is believing that a focus on critical success factors, as a part of a plan based on assumptions and intentions of control and prediction, will solve the personal and organizational issues, eliminate unintended consequences, and ensure success. Instead, he argues for an acceptance of the fundamental unpredictability in such projects, due to the inherent complexity of the mutual relationship between organizational and technological issues. Thus, we will argue that one of the main reasons for the apparent success of the EMR introductions in our two case hospitals is that these factors were accepted as interrelated, handled according to the local contexts, and not treated as a blueprint towards a static future state.

Both the hospital leadership and leading physicians must be committed to the EMR system implementation project for such a project to achieve its goals. The relationship between hospital administrators and clinicians has often been described as problematic (e.g. [18]), where clinicians have been said to be preoccupied with medical excellence and the hospital leaders with budget, economy and accounting (e.g. [19,20]). Often, EMR system introductions have been attributed to the administrative part of the hospital organization. Therefore, one of the greatest challenges, and – as we see it – a prerequisite for the success of a hospital-wide EMR system implementation project, is for both administrative and clinical leadership to be strongly involved and enthusiastic about the project. However, enthusiasm is not enough in itself, and the organization must be prepared to allocate the necessary resources, not only for the technical implementation, but also for the preparation and involvement of relevant parts of the organization before, during and after the implementation.

Without an organization that is willing to undergo changes not much change will take place. We believe that a key factor is to create engagement among the leaders of the clinical departments, something that was achieved in the hospitals involved in this study. From there, the challenge is to establish a common vision and then start committing the rest of the organization. The employees must understand why a change is required, and the concept of envisioning an immediate gain as argued by Berg [21] therefore becomes important. As we were told during the interviews, clinicians generally accepted changes once they saw that the change eased the workload and/or improved the quality of their work. The above point also illustrates the importance of not solely focussing on the EMR system's ability to support individual users conducting single tasks, but also emphasizing the potential effect of the EMR system on clinical workflow – i.e. how the system may facilitate the exchange of responsibilities and tasks between members of the multidisciplinary healthcare team. This approach may however be challenging because of the established professionally centred focus of clinicians [19,20], but still possible as demonstrated especially by hospital North.

### Learning from best practice

Organizations often refer to best practices when introducing new technology such as EMR systems. This is arguably a correct approach, since it may be helpful to gain experiences from relevant successful and unsuccessful projects. Of the hospitals involved in study, the first step in the EMR implementation project in Hospital North was to collect experiences from the corresponding project in hospital South. Valuable insights were obtained by visiting the other hospital. However, they deliberately decided not to make their project a blueprint of that in hospital South, but modified their plans to adapt them to their own unique situation. The EMR system vendor also provided advice based on their experiences as vendor to Hospital South. Based on the lessons from Hospital South, Hospital North formulated a vision of implementing the maximum amount of functionality in the least possible time.

### Achieving collective benefits from organizational change

The contrasting use of the medication module in the two study hospitals is an example of how adjustment of routines in conjunction with the introduction of technology may lead to gains in the chain of processes. Hospital North developed new routines for writing prescriptions, sick leave forms and discharge reports concomitant with the implementation of the EMR system. As a result, the corresponding EMR functions were used extensively and physicians found out that everybody benefit from an updated medication list. Hospital South took a different approach, and chose not to change routines. In our opinion, the unwillingness to change the relevant routines in hospital South largely explains physicians persisting reports of underuse and cumbersomeness when trying to use these EMR functions. Because only a fraction of the physicians used and updated the medication list, physicians could not fully rely on the medication data and therefore did not benefit from re-use of the data when writing encounter notes, prescriptions and discharge summaries. In hospital South, the negative focus on these central EMR system functions also influenced on the physicians' attitude to and reported benefits from the EMR system as a whole. In the absence of beneficial effects of the collective use of the system, the physicians tended to rate their EMR system on the basis of the number of clicks, time to complete tasks and other usability aspects. Thus, we found – in line with Berg [22] – that an EMR system implementation have more profound effects if it also is accompanied by organizational changes. What we have shown is that a relatively small organisational change can have a relatively profound effect.

The need for organizational change may have become more obvious upon the completion of an EMR system implementation project. Accordingly, it may be more difficult to get acceptance for organizational changes during planning of the project. What characterized the respondents from Hospital North was that they described their colleagues as very loyal to the decisions, once these had been made. Thus, when a decision was implemented, clinicians complied by adapting their practices as much as they could. It was considered important, however, that people were encouraged to voice their opinions, both positive and negative, before the decisions were taken. In our opinion, involving clinicians during all phases of the project as well as developing a culture that prepares the department for change are important factors. A culture for change does not imply that decisions must be followed blindly; but rather encourages raising and discussing various alternatives. However, once decisions are made, nobody should try to undermine but instead do their best both for the hospital and their department. Still, since changes are likely to occur also later, openness around the strengths and weaknesses of the decisions should be valued and used to suggest further improvements. In this way an EMR system implementation is not a one-time phenomenon, but a continuous process for IT-supported healthcare activities.

### Poorer support from the EMR system for the work of more experienced physicians

During the interviews two interesting themes emerged; (1) the systems supported freshmen better than experienced physicians, and (2) young users reported to have a different attitude towards the EMR system than more senior users. Both junior and senior physicians agreed that that the EMR system was better at supporting the professional development of junior physicians that those with senior expertise. Senior physicians were supported by the EMR system in their role as supervisors, but not in their role as responsible for quality assessment and quality improvement.

The second theme however might be more complex than it seems. As demonstrated by the American sociologist W. I. Thomas, and later termed the "Thomas theorem," "...what is defined as real is real in its consequences" [23]. In this case, the *idea *that one, as a young physician, should have less problems with the use of computers and information systems, might lead to stubbornness with regard to the task of acquiring new information system skills and patience when it comes to older physicians' asking for IT assistance. A large proportion of the interns and residents in our study had never worked with a paper-based medical record and had therefore never got accustomed with paper-based routines. In contrast, senior physicians that became specialists before the age of EMRs became drilled in paper-based work routines as part of their training. Additionally they seemed to rely more on their own memory instead of that of the computer. As a consequence, they used the computers for fewer tasks than their juniors (according to the juniors). Senior physicians reluctance to the use of computers does therefore not fully explain why they use EMRs less, that old habits die hard (and might be just as effective) and that seniors have slightly different responsibilities at the ward also is part of the explanation.

### The legacy of the paper

As argued, large benefits from implementing an EMR system can only be achieved if they are accompanied by organizational changes. So far, few hospital management teams have dared to impose profound changes in information-handing routines when implementing an EMR system [3,5,7]. This might partly be explained by the legacy of the paper: Even if electronic workflow, ordering of lab results or other functionality has been introduced, the core components of Norwegian EMR systems are still electronic documents containing clinical narratives, bearing strong resemblance to its paper ancestor. Since the paper metaphor has survived the transition from the paper-based medical record to EMRs, paper is still very much alive in Norwegian hospitals. We describe this phenomenon as "paper-thinking".

Our overall impression is that to this day, EMR system implementations in Norway have focused on gradually automating existing manual processes rather than supporting more radical changes. From the perspective of physicians, some complex but crucially important senior clinical tasks have poor support. For these tasks, custom built quality registries and other clinical departmental systems are used [24]. In our opinion, paper-thinking now increasingly is becoming an obstacle to the further development of EMR systems in Norway.

We strongly believe that an EMR that builds on the paper metaphor does not fully leverage the potential benefits of Information and Communications Technologies (ICT). As pointed out by Nelson and Winter (p. 135) [25], "Firms may be expected to behave in the future in ways that resemble the behaviour that would be produced if they simply followed the routines of the past". An EMR system technology that replicates established work routines reinforces this tendency. When clinicians accustomed with paper are left with a choice of using paper or a more cumbersome electronic paper-equivalent, the result is more or less given. At Hospital South, it took almost three years before the majority of physicians started to utilize optional functionalities, even though they were encouraged to use them [7].

## Conclusion

In this study we have demonstrated that many physician users perceive benefits from the EMR systems, but that the legacy of the old paper-based routines and structures still prevails. The challenge now, in our opinion, is to remove paper not just physically, but also to overcome the paper shadow of the past, slowing down the pace of organizational changes. The explicit goal of going paperless should be to streamline processes and improve quality, rather than to save money by not having to maintain a paper archive.

## Competing interests

The author(s) declare that they have no competing interests.

## Authors' contributions

JTL designed the study, participated in all the interviews, transcribed the interviews, analyzed the data and drafted the manuscript; AT participated in the design of the study, helped analyzing the data, revised and helped to draft the manuscript; AF participated in the design of the study, participated in the interviews at hospital South, revised and helped to draft the manuscript. All authors read and approved the final manuscript.

## Pre-publication history

The pre-publication history for this paper can be accessed here:


